# Prevalence of *Chlamydia trachomatis* Genotypes in Men Who Have Sex with Men and Men Who Have Sex with Women Using Multilocus VNTR Analysis-*omp*A Typing in Guangzhou, China

**DOI:** 10.1371/journal.pone.0159658

**Published:** 2016-07-19

**Authors:** Xiaolin Qin, Heping Zheng, Yaohua Xue, Xuqi Ren, Bin Yang, Jinmei Huang, Shujie Huang, Xingzhong Wu, Weiying Zeng, Jiangli Ou, Yinyuan Lan, Sanmei Tang

**Affiliations:** 1 Guangdong Provincial Center for Skin Diseases and STIs Control and Prevention, Guangzhou, Guangdong, China; 2 Department of Laboratory Medicine, Southern Medical University, Guangzhou, Guangdong, China; Cornell University, UNITED STATES

## Abstract

**Background:**

*Chlamydia trachomatis* is one of the most prevalent bacterial sexually transmitted infection in China. Although *C*. *trachomatis* genotypes can be discriminated by outer membrane protein gene (*omp*A) sequencing, currently available methods have limited resolutions. This study used a high-resolution genotyping method, namely, multilocus variable number tandem-repeat analysis with *omp*A sequencing (MLVA)-*omp*A, to investigate the local epidemiology of *C*. *trachomatis* infections among men who have sex with men (MSM) and men who have sex with women (MSW) attending a sexually transmitted diseases (STD) clinic in Guangzhou, China.

**Methods:**

Rectal specimens from MSM and urethral specimens from MSW were collected between January 2013 and July 2014 at the Guangdong Provincial Center STD clinic. The specimens were sent to the laboratory for analyses. All specimens that were tested positive for *C*. *trachomatis* by the commercial nucleic acid amplification tests were genotyped by MLVA-*omp*A.

**Results:**

Fifty-one rectal specimens from MSM and 96 urethral specimens from MSW were identified with *C*. *trachomatis*. One hundred and forty-four of the 147 specimens were fully genotyped by MLVA-*omp*A. Rectal specimens from MSM were divided into four *omp*A genotypes and urethral specimens from MSW into nine genotypes. No mixed infections were found among all specimens. The most frequent genotypes were D, G, J, E and F. All specimens were further divided into 46 types after *omp*A genotyping was combined with MLVA. Genotypes D-8.7.1 and G-3.4a.3 were the most frequent among MSM, whereas genotypes D-3.4a.4, E-8.5.1, F-8.5.1, and J-3.4a.2 were the most frequent subtypes among MSW. The discriminatory index *D* was 0.90 for MLVA, 0.85 for *omp*A, and 0.95 for MLVA-*omp*A.

**Conclusions:**

The most prevalent MLVA-*omp*A genotypes were significantly different between MSM and MSW from Guangzhou, China. Moreover, MLVA-*omp*A represented a more favorable degree of discrimination than *omp*A and could be a reliable complement for *omp*A for the routine subtypes of *C*. *trachomatis*.

## Introduction

*Chlamydia trachomatis* is one of the most common bacterial sexually transmitted infections (STIs) in China [1–3]. Approximately 50% of *C*. *trachomatis* infections in men and 75% of those in women are asymptomatic [4–5]. If untreated, *C*. *trachomatis* infections can cause serious complications, such as urethritis, epididymitis, prostatitis, proctitis, pelvic inflammatory disease infertility and so on [4]. No comprehensive national surveillance on *C*. *trachomatis* infection has been established in China. However, data from 15 sexually transmitted disease (STD) surveillance points in China show that the number of reported cases of *C*. *trachomatis* has been increasing annually. In Guangdong Province, the incidence of reported cases of *C*. *trachomatis* infections increased from 0.5 per population of 100,000 in 2006 to 49.95 per population of 100,000 in 2013 [6, 7]. A large proportion of *C*. *trachomatis* cases remain undiagnosed despite the high number of cases.

*Omp*A genotyping has been used for many years to distinguish *C*. *trachomatis* strains. However, this method identifies only a limited number of distinct subtypes. Various subtypes can persist for a long time within a geographic area, with E, D, and F as the most frequently observed subtypes identified from different regions and countries [8–10]. Given the difficulties in diagnosing persistent or recurrent infections by using only highly conserved *omp*A sequences, other techniques that have high reproducibility and discrimination appear promising. These techniques include the multilocus sequence typing technique based on several housekeeping genes [11, 12] and the multilocus variable number tandem-repeat (VNTR) analysis (MLVA) method, which relies on variation in the number of single nucleotide repeats within three loci (i.e., CT1335, CT1299, and CT1291) [9, 10, 13].

MLVA allows a more precise typing of *C*. *trachomatis* than other methods, as proposed by Pederson *et al*. [13] in 2008 and modified by Wang *et al*. [9] and Bom *et al*. [14]. Labiran *et al*. [15] indicated that MLVA has good stability. The DNA markers for epidemiological studies were selected for typing because of their variability and stability in continuous cell culture; these characteristics allow the collected data to be comparable between studies [15]. Satoh *et al*. used MLVA-*omp*A analysis in 2014 to evaluate the diversity among isolates from venereal specimens collected from clinical settings in the 1980s in Japan [10]. The findings of the studies described above supported the application of the three VNTR loci as markers for *C*. *trachomatis* MLVA-*omp*A genotyping. The data suggested that MLVA-*omp*A genotyping method may be effective as a high-resolution genotyping method in *C*. *trachomatis* isolates.

The objective of this study was to investigate the distribution of *C*. *trachomatis* MLVA-*omp*A genotypes found in rectal infection in MSM and urethral infection in MSW in Guangzhou, China, to c*ompar*e MLVA-*omp*A genotypes between these two populations.

## Materials and Methods

### Ethics statement

The study has been approved by the ethics committee of Guangdong Provincial Center for Skin Diseases and STIs Control and Prevention. All patient data were anonymised. Moreover, the specimens used in this study were all clinical residual specimens, and no personal information was linked to these specimens, we did not need to ask for the patients to provide informed consent.

### Clinical specimen collection and storage

Rectal specimens from MSM were collected using sterile cotton swabs by swabbing the rectal mucosa at a depth of 2 to 3 cm. Urethral specimens from MSW were obtained by inserting the sterile fine cotton swabs 2 to 4 cm into the urethra, gently rotating the swab clockwise for 2 to 3 s to ensure adequate sampling. All specimens were placed in 1.5 ml of sterile physiological saline, stored at -20°C, and processed within one week. All specimens that tested positive for *C*. *trachomatis* by the commercial nucleic acid amplification tests (DaAn Gene Co., Guangzhou, China) were genotyped. These tests are shown below.

### DNA extraction and detection of *C*. *trachomatis*

DNA in clinical specimens (swabs) was extracted by using the Qiagen DNA Isolation Kit according to the protocol for isolation of genomic DNA from bacteria (QIAGEN, Germany). The elution volume was 50 μL. The isolated DNAs were stored at -20°C until use.

Detection of *C*. *trachomatis* by commercial nucleic acid amplification tests was performed in a 20 μL reaction mix prepared as follows: two μL of each extracted DNA and 18 μL of the PCR reaction mixture. Experiments were performed with a Lightcycler2.0 instrument (Roche Diagnostics, Mannheim, Germany) under the PCR conditions: initial denaturation at 93°C for 5 min, followed by 40 cycles of denaturation at 93°C for 30 s and annealing/extension at 55°C for 45 s. In each run a negative and a positive control were included.

### Amplification of VNTR and *omp*A sequences and sequencing

Using the DNA extracts of the two populations above, the three VNTR loci (i.e., CT1335, CT1299, and CT1291, as defined in a previous report) [13, 14] and *omp*A genes were amplified by PCR by using an ABI 7500 fast real-time PCR system (Applied Biosystems, Foster City, CA). The *omp*A gene (VS1-VS2) was amplified by nested PCR using a previously described method in our laboratory [8]. CT1291 was amplified by using CT1291-F and CT1291-R, as described by Pedersen *et al*. [13]. CT1299 and CT1335 were amplified by nested PCR according to a method developed by Bom *et al*. [14] and all primers were synthesized by Life Technologies (AB and Invitrogen) (Life Technologies, Shanghai, China). Primer information is shown in Table 1.

**Table 1:** Primers used for MLVA-*omp*A genotyping for *C*. *trachomatis*.

Region	Primer name	Primer sequence^a^ (5’-3’)	Reference
*Omp*A	*Omp*A-O-F	GCATGCGTATGGGTTACTATGGA	Yang *et al*.[8]
	*Omp*A-O-R	GCRTTRCARAGAACRTTYAAYTC	
	*Omp*A-In-F	ACTTTGTTTTCGACCGTGTTTTG	
	*Omp*A-In-R	GATTGAGCGTATTGGAAAGAAGC	
CT1291	CT1291-F	GCCAAGAAAAACATGCTGGT	Pedersen *et al*.[13]
	CT1291-R	AGGATATTTCCCTCAGTTATTCG	
CT1299	CT1299-O-F	CAACAATCATCACGCCCTCT	Bom *et al*. [14]
	CT1299-O-R	AGCCGCTTTCTCGTTCTAAA	
	CT1299-In-F	CGCTTAAGATTCTCGGAGGTA	
	CT1299-In-R	AAGTCCACGTTGTCATTGTACG	
CT1335	CT1335-O-F	AGTGGGTGTGAAGAACCGTA	Bom *et al*. [14]
	CT1335-O-R	ACCAAACCCTTTTGCAGGAA	
	CT1335-In-F	CGTCCTCTGGAAGGGAATAA	
	CT1335-In-R	TATGCCCCAAGGAAGAGTCA	

^a^In D ⁄ UW-3 ⁄ CX, accession number NC 000117.

The amplified DNA was sent to Life Technologies (AB and Invitrogen) (Life Technologies, Shanghai, China) for DNA sequencing. For the *omp*A, CT1335, and CT1299 genes, the inner forward and reverse primers were used for sequencing; for the CT1291 gene, forward and reverse primers were used for sequencing.

### Genotyping of *C*. *trachomatis* by MLVA-*omp*A sequencing

The obtained *omp*A sequences were compared with sequences on the NCBI database by using BLAST. The VNTR sequences compared the alphabetical sequences to D⁄UW-3⁄CX (NC 000117) and thereafter the assignment of MLVA-types were carried out manually according to the rules described by Pedersen et al. [13], Wang et al. [9], and Satoh et al. [10] (S1 Table).

### Statistical analysis

The discriminatory power of each typing method was calculated by using Hunter and Gaston’s modification of Simpson’s index of diversity [16, 17]. The formula used to define Simpson’s index of diversity (*D*) is: $D=1-\frac{1}{N(N-1)}\sum_{j=1}^{s}x_{j}(x_{j}-1)$, where N is the number of unrelated strains tested, s is the number of different types, and χ_j_ is the number of strains belonging to the j^th^ type. Data obtained from MLVA-*omp*A for specimens were converted into letter and character data sets. A minimum spanning tree (MST) was generated with BioNumerics software (version 7.1, Applied Maths, Sint-Martens-Latem, Belgium).

SPSS 18.0 (IBM) was used in this study for all statistical analyses. Statistical differences in the genotypes between MSM and MSW were analyzed using the Chi-square test. The differences were considered statistically significant when *p* < 0.05.

## Results

### Specimen information

A total of 51 *C*. *trachomatis*-positive rectal specimens from MSM and 96 *C*. *trachomatis* -positive urethral specimens from MSW were obtained at the STD clinic of Guangdong Provincial Center for Skin Diseases and STIs Control and Prevention between January 2013 and July 2014. The age distribution among MSM ranged from 18–39 years of age (median of 24 years of age; S2 Table). The age distribution among the MSW ranged from 18–62 years of age (96, with a median of 34 years of age; S2 Table).

### Genotyping of *C*. *trachomatis* by MLVA-*omp*A sequencing

Direct genotyping from DNA was possible for the two populations of *C*. *trachomatis*-positive specimens. A total of 144/147 (98.0%) *C*. *trachomatis*-positive specimens were fully genotyped by MLVA-*omp*A. Among the 144 fully genotyped specimens, there were 46 different MLVA-*omp*A sequence types.

**Fig 1:**
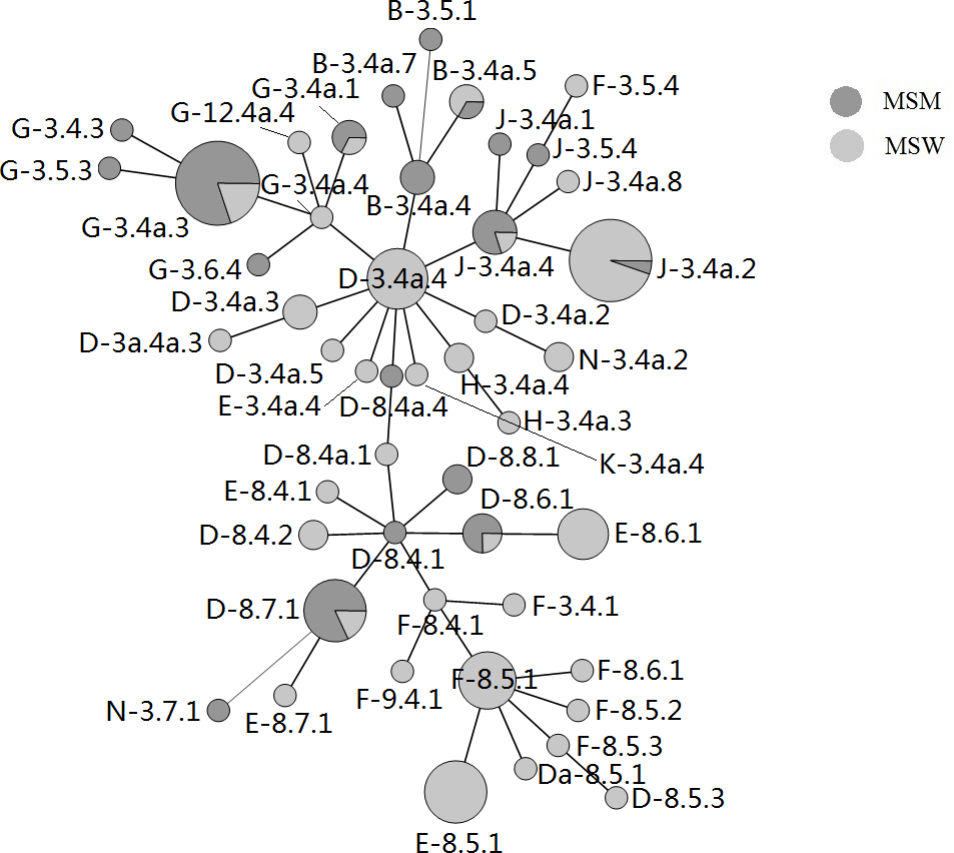
MST of the MLVA-*omp*A genotypes of 147 *C*. *trachomatis* positive specimens from MSM and MSW in Guanghzou between January 2013 and July 2014. Genotypes (MLVA-*omp*A) are indicated with letters within the tree, and each circle denotes a particular MLVA-*omp*A type. Circle size is proportional to the number of specimens. The distance between neighbouring genotypes is expressed as the number of allelic changes. N, non-typeable.

All 147 *C*. *trachomatis* specimens were clustered using a minimum spanning tree based on the MLVA-*omp*A genotypes (Fig 1 and S1 Fig). Among the rectal specimens from MSM, one sample could not be typed by *omp*A genotyping. Of the other 50 specimens, the proportion of genotypes (in ascending order) detected were G, D, J, and B (41.2%, 31.4%, 13.7%, and 11.8% respectively, Table 2). Most genotypes that were indistinguishable by *omp*A gene sequencing could be sub-divided into different divergent types by MLVA typing. The main MLVA types were 3.4a.3 (16/50, 32%) for genotype G, 8.7.1 (9/50, 18%) for genotype D, and type 3.4a.4 for genotypes B (3/50, 6.0%) and J (4/50, 8.0%) (Fig 1). No genotypes for E and F were found.

**Table 2:** Genotype distribution of *C*. *trachomatis* infection in MSM and MSW in Guangzhou.

Populations	No. of *C*. *trachomatis* infection specimens with different *omp*A genotype (%)										Total
	B	D	Da	E	F	G	H	J	K	N	
MSM	6(11.8)	16(31.4)	0	0	0	21(41.2)	0	7(13.7)	0	1(2.0)	51
MSW	2(2.1)	23(24.0)	1(1.0)	21(21.9)	16(16.7)	7(7.3)	3(3.1)	20(20.8)	1(1.0)	2(2.1)	96
Total	8(5.4)	39(26.5)	1(0.7)	21(14.3)	16(10.9)	28(12.2)	3(2.0)	27(18.4)	1(0.7)	3(2.0)	147
χ^2^	6.1	0.9	-	13.0	9.5	24.8	1.6	1.1	-		
*p*	0.014	0.332	-	<0.001	0.002	<0.001	0.202	0.289	-		

N, non-typeable.

Among the 96 urethral specimens from MSW, two specimens could not be typed by *omp*A genotyping. The most common genotypes (in ascending order) were D, E, J, F, and G at 24.0%, 21.9%, 20.8%, 16.7%, and 7.3%, respectively (Table 2). With MLVA-*omp*A sequencing, the main MLVA types were 3.4a.4 (10/94, 10.6%) for genotype D, 8.5.1 (11/94, 11.7%) for genotype E, 3.4a.2 (18/94, 19.1%) for genotype J, 8.5.1 (9/94, 9.6%) for genotype F, and 3.4a.3 (4/94, 4.3%) for genotype G (Fig 1).

All specimens were genotyped successfully by MLVA genotyping. The MLVA types were unique to the *omp*A genotypes in most cases, but there were eleven MLVA genotypes associated with more than one *omp*A genotype (3.4a.1, 3.4a.2, 3.4a.3, 3.4a.4, 3.4a.5, 3.5.4, 8.4.1, 8.5.1, 8.5.3, 8.6.1 and 8.7.1) (S1 Fig). Genotypes A, C, I, and L1-L3 were not found in our specimens, whereas ocular genotype B was found in six cases from MSM and two cases from MSW; Da was in one case from MSW. No mixed infection was found in this research.

### Epidemiology of *C*. *trachomatis* by MLVA-*omp*A sequencing

MSM ≤24 years old represented the majority of *C*. *trachomatis* infection at 58.8% (30/51) followed by MSM between the ages of 25–34 years at 37.3% (19/51). However, MSW between 25–34 and ≥35 years old represented the majority of *C*. *trachomatis* infections at 43.8% (42/96) and 44.8% (43/96), respectively (S2 Table). There was a significantly different age distribution of *C*. *trachomatis* infections between MSM and MSW (χ^*2*^ = 45.3, P<0.001).

The distribution of *omp*A genotypes of B (χ^*2*^ = 6.1, P = 0.014) and G (χ^*2*^ = 24.8, P<0.001) were significantly higher in MSM than MSW, whereas the distribution of E (χ^*2*^ = 13.0, P<0.001) and F (χ^*2*^ = 9.5, P = 0.002) were significantly lower in MSM than MSW. No difference in distribution of D, H and J between MSM and MSW was observed (Table 2).

### Typeability, reproducibility, and discriminatory power

The typeability of the *omp*A gene was 98.0% (144/147). The typeability of VNTRs was 100.0% (147/147). All PCR and sequencing results could be reproduced. The discriminatory power (*D*) was calculated using 147 epidemiologically unrelated specimens. The individual *D* s of specimens from MSM were *D*_*omp*A_ = 0.71, *D*_MLVA_ = 0.86, and *D*_*omp*A-MLVA_ = 0.87. The individual *D* s of specimens from MSW were *D*_*omp*A_ = 0.82, *D*_MLVA_ = 0.87, and *D*_*omp*A-MLVA_ = 0.93. For all specimens, the individual *D*s were *D*_*omp*A_ = 0.85, *D*_MLVA_ = 0.90, and *D*_*omp*A-MLVA_ = 0.95.

## Discussion

In this study, one new variant code (bolded) of VNTR was identified in genotype J (CT1291, code 8: AAAATGGTCT-**7C**-TATTG) from MSW; this result obeyed Satoh *et al*.’s rules (CT1291, code 8b: AAAAT**A**GTCT**A**-**7C**-TATTG) [10]. Some other new variant codes (bolded) of VNTR identified in these studies of Wang *et al*. [9] and Satoh *et al*. [10] were also found in our study (i.e., (CT1335, code 12: GAAAAA**A**G-**8T9A**-GCTTTTGT) and (CT1291, code 7: AAAATGGTCT-**12C**-TATTG)). Some modified variant codes (bolded) were also identified in the study (i.e., (CT1335, code 3a: GAAAAA**A**G-**10T8A**-GCTTTTGT) and (CT1299, code 4a: TTTTTATTCT-**10C-T3C**-ATCAAA)) when using the rules of assignment of Wang *et al*. [9] (S1 Table).

The genotyping of *C*. *trachomatis* is vital for epidemiology studies. In our study, the most prevalent *omp*A genotypes in MSM were G (41.2%) and D (31.4%) followed by J (13.7%) and B (11.8%) in Guangzhou, which showed a close resemblance to that of MSM in Shenzhen [18], Sweden [19] and Amsterdam [20]. However, in Northern Spain, the distribution of genotypes in MSM was E (37. 5%) followed by G (25%), D (12. 5%), J (10%) and L2b (5%) [21], which was just partly similar to Guangzhou. Notably a high prevalence of L2b had been found in Northern Spain [21], Amsterdam [20], and Brighton [22] before, but no case of LGV was found in Shenzhen [18] and Guangzhou, China.

We also found that there were distinct distributions of *C*. *trachomatis* genotypes for MSM and MSW. MSM were mainly infected by genotypes G, D, J, and B, whereas MSW were mainly infected by genotypes D, E, J, and F, which bore a close resemblance to that of MSW in Jiangsu, Guangxi, Hainan (China) [23] and Amsterdam (Netherland) [20]. In MSM, the age distribution of *C*. *trachomatis* infections was significantly lower than MSW (χ^2^ = 45.3, P<0.001). The difference may be caused by the different sexual activities in these two populations.

Both MSM and MSW populations share the high prevalence of D and J genotypes. However, the MVLA types show greater difference in the *omp*A genotypes (Fig 1 and S1 Fig). The data of our study shows that the epidemic trend of E-8.5.1 is similar to Amsterdam [20], Japan [10] and Brighton [22]. However, some other MLVA-*omp*A types show a different epidemic trend to compare with these different areas of the world. Interestingly, these results represent clusters of cases that might indicate specific sexual networks in terms of lines of transmission in Guangzhou, China.

This study showed that the circulating *C*. *trachomatis* strains in southern China have higher genetic diversity than what can be measured by *omp*A-based genotyping alone. This result revealed a diversity measured by differences in MLVA. However, this result also showed that the diversity of MLVA types were unique to specific *omp*A genotypes. A diversity of *omp*A types was also found within certain common MLVA types (S1 Fig), which might imply *omp*A mobility. For example, MLVA type 3.4a.2 was distributed between *omp*A types D and J; MLVA type 3.4a.3 was distributed between *omp*A types D, G, and H, which strongly improved the discriminatory power. The discriminatory index increased from 0.85 (*D*_*omp*A_) to 0.95 (*D*_*omp*A-MLVA_), which was similar to previous reports [9, 10, 13–15]. Thus, the determining factors of the chains of transmission of *C*. *trachomatis* might be found. Clinical specimens with genotypes E, F, and D exhibited the same or similar genetic patterns as reference strains (e.g., E/ IU-1579, F/IU-1607, and D/IU-FQ1053) by *omp*A analysis. Those with genotypes D, E, F, and G exhibited the same or similar genetic patterns as isolates in a previous report in Japan [10].

The potential limitation of this research was that all specimens were obtained from men in the researchers’ hospital. The lack of female specimens might present a different epidemic trend. A large size of *C*. *trachomatis* specimens from different areas or hospitals need to be used for further research. In conclusion, this study was the first time that the MLVA-*omp*A typing method was used to analyze the epidemiology of *C*. *trachomatis* from MSM and MSW in Guangzhou, China. The MLVA-*omp*A high-resolution genotyping system can be successfully applied to specimens from MSM and MSW. The analysis of MLVA-*omp*A genotyping produced epidemiologic data about *C*. *trachomatis* infection and transmission that were far superior to traditional *omp*A typing in terms of resolution, particularly of the globally predominant genotypes E, D, and F.

## Supporting Information

S1 Fig MST of the MLVA genotypes of 147 *C*. *trachomatis* positive specimens from MSM and MSW in Guanghzou between January 2013 and July 2014. Genotypes (MLVA*-omp*A) are indicated with letters within the tree, and each circle denotes a particular MLVA type. Circle size is proportional to the number of specimens. The distance between neighbouring genotypes is expressed as the number of allelic changes. N, non-typeable.
(TIF)

S1 Table VNTR sequence analysis and description of previously unseen VNTR types. ^a^VNTR region is shown in bold. Flanking region variations are shown in bold and underlined.
(DOCX)

S2 Table Age distribution of *C*. *trachomatis* infection from MSM and MSW. (DOCX)
