# Supplementary figures and images for: Prevalence of Chlamydia trachomatis Genotypes in Men Who Have Sex with Men and Men Who Have Sex with Women Using Multilocus VNTR Analysis-ompA Typing in Guangzhou, China
Source: PLoS One. 2016 Jul 19;11(7):e0159658. doi: 10.1371/journal.pone.0159658 (PMC4951006; doi:10.1371/journal.pone.0159658)

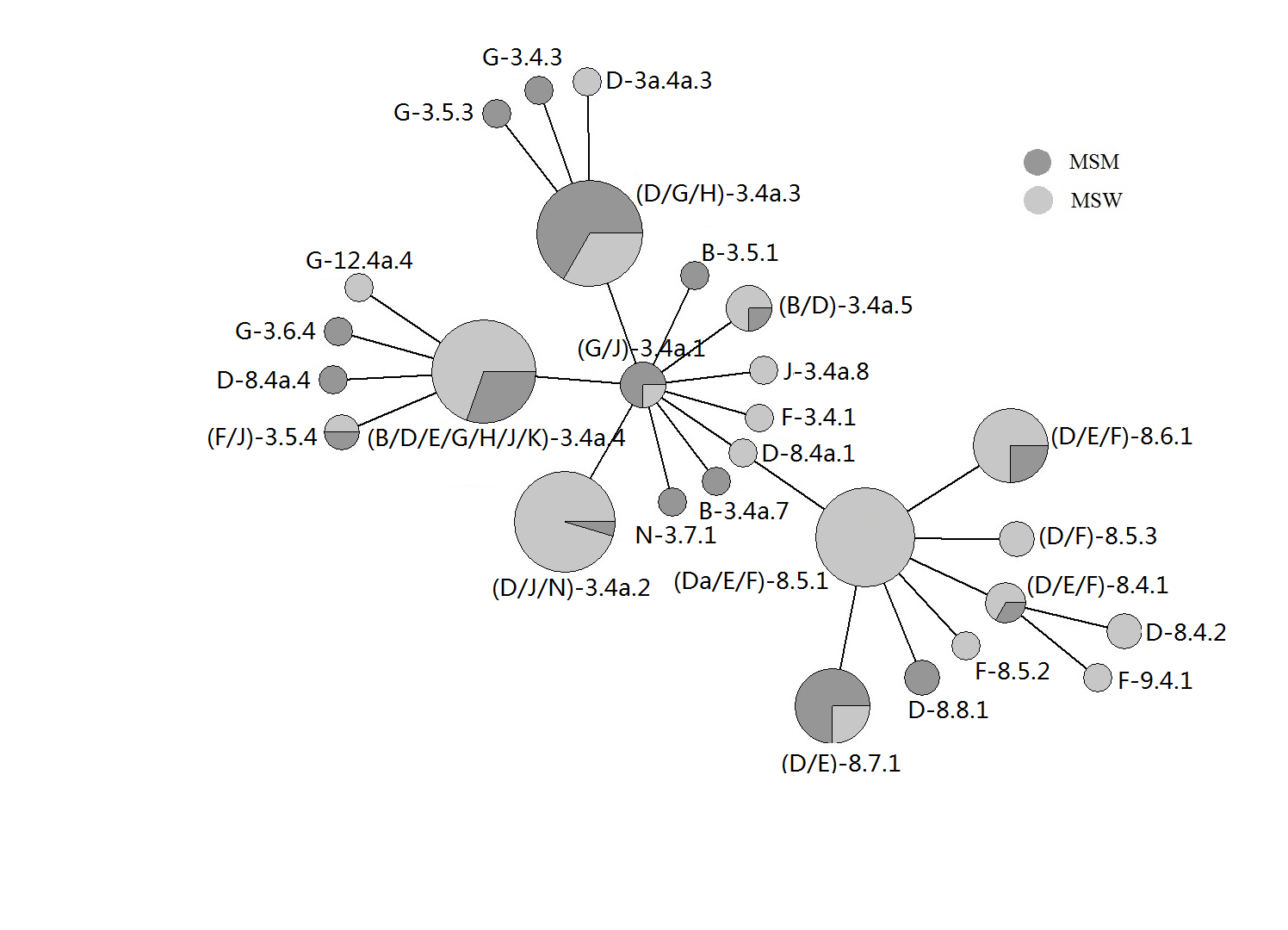

Supplement: S1 Fig — Genotypes (MLVA-ompA) are indicated with letters within the tree, and each circle denotes a particular MLVA type. Circle size is proportional to the number of specimens. The distance between neighbouring genotypes is expressed as the number of allelic changes. N, non-typeable. (TIF) [file pone.0159658.s001.tif]
