# Supplementary material for: Prevalence of Chlamydia trachomatis Genotypes in Men Who Have Sex with Men and Men Who Have Sex with Women Using Multilocus VNTR Analysis-ompA Typing in Guangzhou, China
Source: PLoS One. 2016 Jul 19;11(7):e0159658. doi: 10.1371/journal.pone.0159658 (PMC4951006; doi:10.1371/journal.pone.0159658)
Supplement: S1 Table — aVNTR region is shown in bold. Flanking region variations are shown in bold and underlined. (DOCX) [file pone.0159658.s002.docx]

**S1 Table. VNTR sequence analysis and description of previously unseen VNTR types**

| VNTR type code (including new and modified) | CT1335 Variants^a^ |
| --- | --- |
| 1 | GAAAAAG-**9T8A**-GCTTTTGT |
| 3 | GAAAAAGG-**10T8A-**GCTTTTGT |
| 3a (modified) | GAAAAA**A**G-**10T8A-**GCTTTTGT |
| 5 | GAAAAAG-**11T8A**-GCTTTTGT |
| 6 | GAAAAAGG-**12T7A**-GCTTTTGT |
| 7 | GAAAAAGG-**12T8A**-GCTTTTGT |
| 8 | GAAAAAGG-**13T7A**-GCTTTTGT |
| 9 | GAAAAAGG-**13T6A**-GCTTTTGT |
| 11 | GAAAAA**A**G-**7T9A**-GCTTTTGT |
| 12 | GAAAAA**A**G-**8T9A**-GCTTTTG |
| 13 | GAAAAAGG-**9T9A**-GCTTTTGT |
| 14 | GAAAAAGG**-6T7A-**GCTTTTGT |
|  | CT1299 Variants^a^ |
| 1 | TTTTTATTCT**-7C-**ATCAAA |
| 2 | TTTTTATTCT**-8C-**ATCAAA |
| 3 | TTTTTATTCT**-9C-**ATCAAA |
| 3a (modified) | TTTTTATTCT**-9C-T3C**-ATCAAA |
| 4 | TTTTTATTCT**-10C-**ATCAAA |
| 4a (modified) | TTTTTATTCT**-10C-T3C**-ATCAAA |
| 4b (modified) | TTTTTATTCT**-CT-10C-T3C**-ATCAAA |
| 5 | TTTTTATTCT**-11C-**ATCAAA |
| 5a (modified) | TTTTTATTCT**-11C-T3C-**ATCAAA |
| 6 | TTTTTATTCT**-12C-**ATCAAA |
| 7 | TTTTTATTCT**-13C-**ATCAAA |
| 8 | TTTTTATTCT**-14C-**ATCAAA |
| 9 | TTTTTATTCT**-3C2T-6C-**ATCAAA |
|  | CT1291 Variants^a^ |
| 1 | AAAATGGTCTA**-6C-**TATTG |
| 2 | AAAATGGTCT**-8C-**TATTG |
| 2a (modified) | AAAATGGTCT**A-8C-**TATTG |
| 2b (modified) | AAAAT**A**GTCT**A-8C-**TATTG |
| 3 | AAAATGGTCT**-9C-**TATTG |
| 3b (modified) | AAAAT**A**GTCT**A-9C-**TATTG |
| 4 | AAAATGGTCT**-10C-**TATTG |
| 5 | AAAATGGTCT**-11C-**TATTG |
| 6 | AAAATGGTCT**-CT-5C-**TATTG |
| 7 | AAAATGGTCT**-12C-**TATTG |
| 8 (new) | AAAATGGTCT**-7C-**TATTG |
| 8b (modified) | AAAAT**A**GTCT**A-7C-**TATTG |
| 9 | AAAATGGTCT**-14C-**TATTG |

^a^VNTR region is shown in bold.

Flanking region variations are shown in bold and underlined.
