# Supplementary material for: Prevalence of Chlamydia trachomatis Genotypes in Men Who Have Sex with Men and Men Who Have Sex with Women Using Multilocus VNTR Analysis-ompA Typing in Guangzhou, China
Source: PLoS One. 2016 Jul 19;11(7):e0159658. doi: 10.1371/journal.pone.0159658 (PMC4951006; doi:10.1371/journal.pone.0159658)
Supplement: S2 Table — (DOCX) [file pone.0159658.s003.docx]

**S2 Table. Age distribution of *C. trachomatis* infection from MSM and MSW**

| Age group (y) | No. with rectal *C. trachomatis* infection in MSM (%) | No. with urethral *C. trachomatis* infection in MSW (%) | χ*^2^* | *p* |
| --- | --- | --- | --- | --- |
| ≤24 | 30 (58.8) | 11 (11.5) | 45.3 | ＜0.001 |
| 25-34 | 19 (37.3) | 42 (43.8) |  |  |
| ≥35 | 2 (3.9) | 43(44.8) |  |  |
| Total | 51 | 96 |  |  |
